# External Use of Salicylic Acid

**Published:** 1877-01

**Authors:** 


### ARTICLE V.

### *External Use of Salicylic Acid.*

This remedy seems destined, if we judge from the great variety of therapeutical purposes to which it has already been successfully applied, to reach a high stand in the Materia Medica, and displace many from the positions they have long occupied. Its value as an antiseptic is likely to be the most conspicuous of its merits. Applied to the skin it appears to arrest certain morbid processes, deodorizes foul ulcerating surfaces, re-establishes actions of health, and rapidly cures after other approved measures have failed to be of service. One of the effects of this acid is undoubtedly that of an irritant. If some of the powder be mixed with water and swallowed, disagreeable, smarting sensations in the throat are experienced until it has been washed down by a succeeding draught of water unmixed. Furthermore, we believe it causes a disturbance of the bowels, and provokes flatulency and a tendency to diarrhœa. This, at least, is our own experience with it when taken in doses not exceeding five to ten grains. Others, who have given it in all quantities, large and small, have omitted any notice of such action, from which fact it may be concluded that such disturbance has not occurred in their practice. The satisfactory results obtained from its external use by Dr. Ogilvie Will, Assistant Surgeon to the Aberdeen Royal Infirmary, have led him to devise formulæ for applying it to the skin. With those formulæ he is so well satisfied that he has given them currency in the London *Lancet.* Some we republish in the *Druggists' Circular.* It is convenient to have them at hand whenever they happen to be needed.

Dr. Will says:—To a surface where it can be accurately applied and where the suppurative discharge was slight though I have tried it with excellent effect where it was profuse, the cerate suggested by Prof. Lister for rodent ulcer— salicylic taking the place of boracic acid—is a nice application. The formula is—

| | | |
|---|---|---|
| I. | Salicylic acid, - | ½ to 1 drachm. |
| | White Wax, - - | 1 " |
| | Paraffine, - - - | 2 drachms. |
| | Almond oil, - - | 2 " |

Meet and rub up in a heated mortar. It should be spread on strips of muslin or fine linen.

| | | |
|---|---|---|
| II. | Sperm oil - - | 1½ drachms. |
| | Oil of theobroma - | 5½ " |
| | Salicylic acid, - | ½ to 1 drachm. |

This forms a thick paste, which should be thickly spread on lint. The heat of the surface acting on the cocoa butter, a disfusible ointment is formed, which is a suitable application when it is desired to have the discharge thoroughly saturated with the antiseptic. Another ointment, less acted on by the warmth of the body, may be made by taking:

| | | | |
|---|---|---|---|
| III. | Sperm oil, | | |
| | Paraffine, of each, - | 1½ | drachms. |
| | Oil of theobroma - | 2 | " |
| | Oil of almonds, - | 1 | " |
| | Salicylic acid, - | ½ to 1 | " |

A very simple and most useful ointment and one which answers admirably in some affections of the skin, is formed of half a drachm to a drachm of the acid to seven drachms of simple ointment. Though neither watery nor oily preparations of this acid belong to what is termed elegant pharmacy, yet a liniment of salicylic acid and olive oil will be found of much efficacy in burns; as an example I may cite a case of burn, involving the upper arm and nearly the whole back of a child aged seven, when I recently employed it. Carron oil had been applied; and when my advice was requested, two days after, free suppuration had taken place, accompanied by great fetor. Lint soaked in olive oil and salicylic acid—twelve drachms of the acid to sixteen ounces of oil—was prescribed. The result was most satisfactory; the fetor disappearing, the suppuration rapidly decreasing, the pain greatly abating, and kindly healing action taking place. In less than a fortnight the whole surface was healed, with the exception of two small spots covered by healthy granulations, to which red lotion was applied. I have since found that a much weaker oily solution (two drachms of the acid to eight ounces of oil) was all sufficient in a case of severe scald of the foot lately under treatment.

Though I stated in the outset that I did not intend entering on details of cases, yet I cannot refrain from urging its use in eczematous affections, for nowhere is its beneficial action more marked than in them. In the eczema of children, more especially in that found affecting the head and face, it is far superior to any remedy I have yet employed; it was, in fact, the results attending the use of salicylic acid in a case of the kind that first convinced me of its value, and I have since found that others have formed a like estimate of it in similar cases. A child, aged eighteen months, had been under my care for many weeks with a patch of eczema rubrum on the cheek; I had tried all kinds of local application, and had given arsenic and iron wine internally- but with no avail; for though at times it seemed to be giving way to treatment, a fit of itching, I suppose, had caused the child to rub it, and increased irritation always followed. I had not before used salicylic acid, and had but little faith in good accruing from its exhibition, but as a last resource I prescribed an ointment containing one drachm of acid to an ounce of lard. When I next visited the child, a few days after, the patch had nearly healed, and very shortly after it was perfectly whole. In another case the result was almost equally striking, but here it was the first application used. A child, aged six months, had a patch of E. impetiginodes on the occiput; it had first appeared about a month before. As some parts were thickly covered by crusts, a poultice was used to detach them, and then salicylic ointment was rubbed in. In six days every vestige of disease had disappeared. A brother and sister, aged three and five, were the subjects of eczema narium of short standing. Salicylic ointment was applied on a Friday, and on the following Monday the surfaces were perfectly healed. It is unnecessary to occupy space by entering on details of other cases, but I may simply say that in very many I have found it to answer admirably. —*Druggists' Circular.*

